# Long-term Prognostic Value of Major and Minor ECG Abnormalities in Latent Keshan Disease With Suspect Chronic Keshan Disease

**DOI:** 10.2188/jea.JE20130180

**Published:** 2014-09-05

**Authors:** Yanhe Zhu, Bingqi Lai, Xiaolin Niu, Jin Wei, Wuhong Tan, Xinfeng Wang

**Affiliations:** 1 Institute of Endemic Disease, Key Laboratory of Environment and Gene Related to Diseases of Ministry Education, School of Medicine, Xi’an Jiaotong University, Xi’an, China; 2 Department of ICU, The Tumor Hospital of Shaanxi Province, Xi’an, China; 3 Department of Cardiology, Second Affiliated Hospital, School of Medicine, Xi’an Jiaotong University, Xi’an, China; 4 Cardiovascular Research Center, Department of Physiology and Pathophysiology, School of Medicine, Xi’an Jiaotong University, Xi’an, China

**Keywords:** Keshan disease, selenium, electrocardiogram

## Abstract

**Objective:**

This study aims to determine whether baseline electrocardiography (ECG) abnormalities, the appearance of new ECG abnormalities, or other clinical characteristics are associated with increased rates of progression to chronic Keshan disease (KD) among patients with latent KD.

**Methods:**

Four hundred and fourteen new latent KD patients from a monitored population in China were diagnosed and then followed for 10 years. Baseline and 10-year ECG abnormalities were classified according to the Minnesota Code as major and minor. Using Cox proportional hazards regression models, the addition of ECG abnormalities to traditional risk factors were examined to predict chronic KD events.

**Results:**

In 414 latent KD patients with ECG abnormalities, 220 (53.1%) had minor and 194 (46.9%) had major ECG abnormalities. During the follow-up, 92 (22.2%) patients experienced chronic KD events; 32 (14.5%) and 60 (30.9%) of these chronic KD events occurred in the minor and major ECG abnormalities groups, respectively. After adjustment for baseline potential confounders, the hazard ratios and 95% confidence intervals (CIs) for progression to chronic KD in latent KD patients with major ECG abnormalities versus those with minor ECG abnormalities was 2.43 (95% CI 1.58–3.93).

**Conclusions:**

Major ECG abnormalities and new ventricular premature complex abnormalities that occurred during the follow-up were both associated with an increased risk of progression to chronic KD. Atrial fibrillation and right bundle branch block with left anterior hemiblock are the most strongly predictive components of major ECG abnormalities. Depending on the model, adding ECG abnormalities to traditional risk factors was associated with improved risk prediction in latent KD.

## INTRODUCTION

Keshan disease (KD) is an endemic cardiomyopathy with unknown etiology that was first found in Keshan County, Heilongjiang Province, China, during winter in 1935, when a violent and tragic outbreak that resembled a plague^1^ occurred. Thus, the disease was named after the place.^2^ Later, there were similar reported cases of KD in Nagano Prefecture of Japan and in the northern mountains of North Korea in the 1950s.^3^ Clinically, KD patients are divided into four categories on the basis of the onset of attack, clinical features, and heart function.^4^^–^^6^ These categories are acute, subacute, chronic, and latent. Few cases of acute or sub-acute KD have been reported in recent years, and chronic and latent KD patients are mainly found in KD endemic areas.^7^ Chronic KD is characterized by severe cardiomyopathy, which is usually manifested by congestive heart failure and varying degrees of pathological changes. Few effective treatment measures have been discovered for chronic KD to date. The five-year mortality rate for chronic KD remains high.^8^ A number of latent KD cases develop into chronic KD and tend to be the primary source of chronic KD.^9^ However, most latent KD cases are asymptomatic and are usually untreated. Therefore, the identification of latent KD cases, which have a high risk of developing into chronic KD, is crucial.

Electrocardiography (ECG) is the most common noninvasive test used for diagnosing latent KD. ECG has high sensitivity in latent KD; thus, risk prediction that incorporates ECG is suitable for such cases. However, few studies have examined the improvement of chronic KD risk prediction based on identification of abnormalities using ECG in latent KD.

In the present study, a 10-year follow-up of latent KD patients was conducted to determine whether baseline major and minor ECG abnormalities are associated with incident chronic KD events.

## METHODS

### Study population

All newly diagnosed latent KD patients with ECG abnormalities were enrolled from monitored populations in Xunyi and Huangling Counties, Shaanxi Province, China, from March 1997 to December 2012. A total of 448 latent KD patients were visited at the beginning of the follow-up. All patients were diagnosed according to the National Criteria for Diagnosis of Keshan Disease. Latent KD must satisfy the following conditions: 1) geographic distribution in endemic areas—newcomers to an endemic area must have adopted a local diet and lifestyle for at least three months; 2) existing symptoms and signs of heart disease and/or ECG abnormalities; 3) exclusion of other heart diseases. Latent KD usually shows mild heart enlargement with normal heart function (New York Heart Association Class I), and a number of such cases are asymptomatic. Almost all patients with latent KD exhibit abnormal ECG changes.^10^ All subjects with conditions such as accompanying pulmonary fibrosis and malignant chronic wasting diseases—including cancer, renal disorders, and hepatocirrhosis—as well those aged over 65 years, were excluded from the study. All participants gave informed consent during each examination, and the present study protocol was approved by the Human Ethics Committee of Xi’an Jiaotong University.

Medical records were created for all new cases examined at baseline; the information included medical history, physical examination, 12-lead ECG, and chest radiography. The examinations during the follow-up in late April every year only included a physical examination and an electrocardiogram. The participants were contacted via telephone during the remainder of the examination period. The suspects with chronic KD underwent additional chest X-ray and echocardiography. Fasting venous blood samples were collected from the new cases, and whole blood selenium concentration and glutathione peroxidase (GPx) activity were measured. If a participant reported an interim hospitalization and a chronic KD event was suspected during the follow-up, then the hospital records were obtained and reviewed. The hospital records of the participants with cardiovascular diagnoses were reviewed by a cardiologist, who then gave a diagnosis of definite or possible chronic KD.

### ECG data

Standard 12-lead ECGs were annually recorded at baseline in all of the latent KD patients using strictly standardized procedures; the patients were in resting supine position. ECG technicians were trained to decrease chest electrode placement errors, thereby decreasing inter-technician variability. All of the ECGs were read by two cardiologists, and discordant results were adjudicated by a senior cardiologist. ECGs were coded according to the Minnesota Code (MC).^11^^,^^12^

Electrocardiographic abnormalities were divided into major and minor abnormalities on the basis of the MC. The criteria for major prevalent ECG abnormalities were any of the following: complete bundle branch block or intraventricular block (MC 7-1-1, 7-2-1, 7-4, or 7-8), left ventricular hypertrophy (MC 3-1), atrial fibrillation or atrial flutter (MC 8-3), major ST-T changes (MC 4-1, 4-2, 5-1, and 5-2), major AV conduction abnormalities (MC 6-1, 6-2, 6-4, and 6-8), major QT prolongation (QTi ≥ 116%), and supraventricular tachycardia (MC 8-4-2). The criteria for minor prevalent ECG abnormalities were minor ST-T changes (MC 4-3, 4-4, 5-3, and 5-4), premature beats (MC 8-1-1, 8-1-2, 8-1-3, and 8-1-5), incomplete (left and right) bundle branch block (MC 7-3, 7-6, and 7-7), sinus tachycardia or bradycardia (MC 8-7, 8-8), minor QT prolongation (QTi ≥ 112%), short or long PR interval (MC 6-3, 6-5), left or right axis deviation (MC 2-1, 2-2), and other minor arrhythmias. Participants with both major and minor abnormalities were classified as having major abnormalities. Participants with no ECG abnormalities were classified as having no abnormalities, and their ECGs were considered normal.

### Chronic KD events

Chronic KD was diagnosed according to the National Criteria (GB 17021-1997, Ministry of Health, China).^10^ Follow-up time was defined as the time from the baseline visit until the chronic KD event date (for participants who had an event) or the end of the 10-year study (for participants who did not have any events).

### Measurement of whole blood selenium concentration and GPx activity

Whole-blood selenium concentrations were measured using a fluorometric method and hydride atomic fluorescence spectrometry (AFS 2201A; Wantou Co., Beijing, China).^13^ The whole-blood GPx activity was assayed by a standard coupled spectrophotometric method using a Micro-Ultraviolet Spectrophotometer (Nanjing Jiancheng, Beijing, China) at 412 nm. One activity unit was defined as a decrease in concentration by 1 µmol/L of glutathione in the reaction system per 8 µL whole blood reacted at 37 °C for 5 min after subtracting non-enzymatic reaction. Results were expressed as U/g Hb.

### Other covariates

Covariates, including age, sex, cigarette smoking, family history of KD, body mass index (BMI), blood pressure, heart rate, initial cardiothoracic ratio, and new-onset of hypertension diagnosed during the following period, were defined by self-reporting. The use of antihypertensive medications and a measured blood pressure with a systolic blood pressure of 140 mm Hg or higher, a diastolic blood pressure of 90 mm Hg or higher, or both were also self-reported by the patients. The appearances of new ventricular premature complexes (VPCs), which were secondary to the primary ECG abnormality during the follow-up and coexisted with it, were also observed.

### Statistical analysis

In this study, statistical analysis was performed using SPSS 13.0 software (IBM, Armonk, NY, USA). Continuous variables were described by mean ± SD. Comparisons between latent KD groups were performed using χ^2^ test for categorical variables and Student’s *t*-test for continuous variables. A value of *P* < 0.05 was considered significant.

Cox proportional-hazards regression analysis was applied to identify the independent predictors of chronic KD events by including the ECG date. The variables tested were age, sex, initial cardiothoracic ratio, BMI, blood pressure, heart rate, cigarette smoking, family history of KD, plasma selenium level, hypertension, VPCs, and minor and major ECG abnormalities. A value of *P* < 0.05 in the univariate analysis was required for inclusion in the multivariate analysis. Multivariate analysis was performed in a stepwise fashion. At each step, variables were removed from the model until all the remaining variables were significantly independent (*P* < 0.05).

Cumulative event rates for each group were obtained using Kaplan-Meier method for all the chronic KD events and compared with Wilcoxon log-rank test in all the patients with low to intermediate and high pre-test likelihood of chronic KD. Statistical significance was defined as *P* < 0.05.

## RESULTS

### Electrocardiograms and other baseline data

Among the 448 latent KD patients, 28 with missing data for any of the risk factors were excluded. Six participants with sudden death were also excluded. The deadline for follow-up was December 2012. The final sample for the analyses of baseline ECG abnormalities consisted of 414 participants. The characteristics of the latent KD patients are shown in Table 1. At baseline, 264 (63.8%) of the 414 latent KD patients with ECG abnormalities were women, and mean age of all of these participants was 39.7 ± 13.3 years. Of the 414 latent KD patients, 220 (53.1%) possessed minor and 194 (46.9%) possessed major ECG abnormalities.

**Table 1.** Baseline characteristics of the study population

Characteristics	Number (%) of participants			*P* value

	All (*n* = 414)	Minor ECG abnormality (*n* = 220)	Major ECG abnormality (*n* = 194)	
Age, mean (SD), y	39.7 (13.3)	37.6 (13.5)	42.1 (12.7)	<0.05
Women	264 (63.8)	159 (72.2)	105 (54.1)	<0.01
Family history of KD	50 (12.1)	28 (12.7)	22 (11.3)	0.25
Plasma Se level (SD), µg/L	67.7 (19.8)	68.9 (20.8)	66.4 (18.9)	0.19
Smoker	106 (25.6)	30 (13.6)	76 (39.2)	<0.01
Heart rate	74.3 (5.3)	74.1 (5.2)	74.1 (5.5)	0.56
Body mass index (kg/m^2^)	20.7 (1.7)	20.5 (1.8)	20.9 (1.5)	<0.05
Systolic blood pressure (mm Hg)	116.9 (11.0)	114.8 (11.1)	119.3 (10.4)	0.20
Diastolic blood pressure (mm Hg)	74.9 (5.6)	73.7 (5.6)	76.2 (5.4)	0.08
Initial cardiothoracic ratio	0.5 (0.06)	0.5 (0.05)	0.5 (0.06)	0.51

ECG, electrocardiogram; KD, Keshan disease; SD, standard deviation; Se, selenium.

During the follow-up (112.9 ± 17.5 months), 92 (22.2%) cases developed into chronic KD. Thirty-two (14.5%) and 60 (30.9%) chronic KD cases were diagnosed in the minor and major ECG abnormalities groups, respectively.

### Correlation of GPx enzyme activity with selenium blood concentration

**Figure 1.**
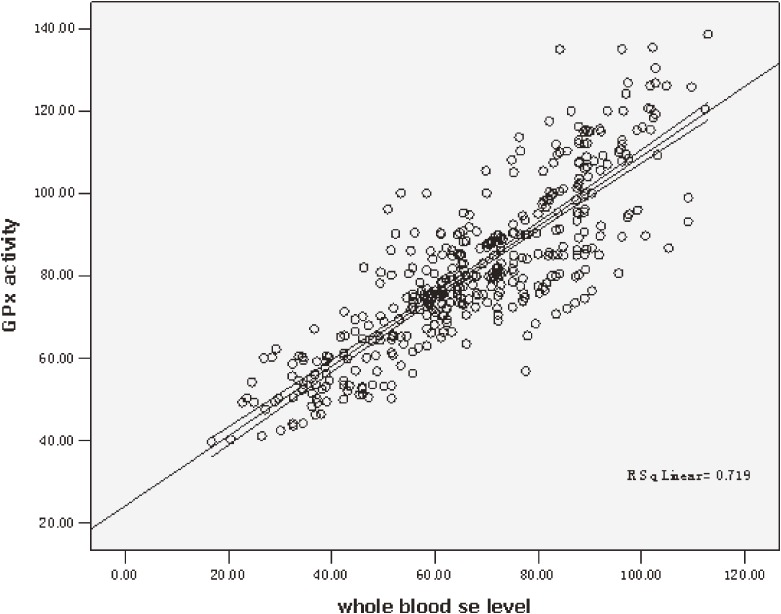
GPx activity and whole-blood selenium (Se) concentration in latent KD patients. Scatter plot and regression results of correlation between GPx activity and whole-blood selenium concentration for all participants. Equation: GPx activity = 24.28 + 0.848 Se level (*r* = 0.719, *P* < 0.01).

Whole-blood selenium concentration at baseline ranged from 16.70 to 112.90 µg/L, with no significant difference in terms of sex and age, and the mean value was 67.69 ± 19.8 µg/L, which is lower than that in Kłapcińska’s report (80–250 µg/L) for the populations in areas where KD is non-endemic.^14^ GPx activity linearly increased with increasing blood selenium concentration (Figure 1).

**Figure 2.**
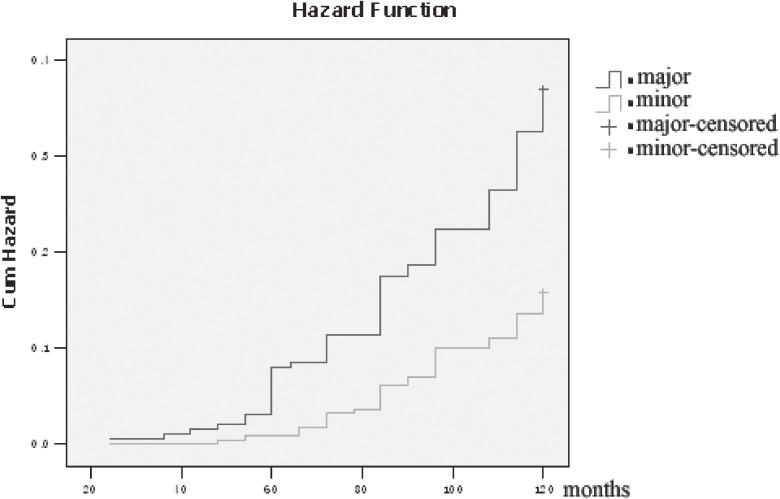
Kaplan-Meier estimates of chronic KD cumulative hazard over time of major vs minor ECG abnormalities.

The Kaplan-Meier estimates of chronic KD cumulative hazard over time for latent KD cases with minor ECG abnormalities versus major ECG abnormalities were calculated, and the results are shown in Figure 2, which shows a significant difference in chronic KD outcomes (log-rank *P* < 0.01) between the two groups. Major ECG abnormalities at baseline were associated with an increased risk of chronic KD.

After adjustment for age, sex, initial cardiothoracic ratio, family history of KD, BMI, blood pressure, heart rate, smoking, and plasma selenium level, the hazard ratios (HRs) and 95% confidence intervals (CIs) for progression to chronic KD in latent KD patients with major ECG abnormalities versus those with minor ECG abnormalities was 2.30 (95% CI 1.44–3.67) at baseline.

### Follow-up ECGs

A total of 389 latent KD patients had a persistent ECG abnormality during the follow-up. Among patients with latent KD and an ECG abnormality, 20 had new VPC abnormalities during the follow-up, and 11 of these latent KD patients with persistent abnormalities (5 with minor and 6 with major ECG abnormalities) had ECG results that changed to normal for less than 2 years before returning to abnormal status. Three latent KD cases with major ECG abnormalities progressed to chronic KD during the follow-up. The ECGs of the other 25 latent KD patients (20 with minor and 5 with major ECG abnormalities) became normal within 7 years and maintained normality for over 3 years. Thirty-six latent KD patients (15 with minor and 21 with major ECG abnormalities) had hypertension during the follow-up.

After adjustment for baseline age, sex, initial cardiothoracic ratio, family history of KD, BMI, blood pressure, heart rate, smoking, and plasma selenium level, secondary ECG abnormalities and hypertension during the follow-up were associated with an increased risk of subsequent progression to chronic KD (for new VPC abnormalities: HR 2.05, 95% CI 1.09–3.87; for hypertension: HR 2.23, 95% CI 1.16–4.28), as shown in Table 2.

**Table 2.** Hazard ratios for incidence of chronic KD events in latent KD patients

Variable	Wald	*P*	Exp (β)	95% CI
New VPC	5.00	<0.05	2.05	1.09–3.87
Plasma Se level	55.64	<0.01	0.95	0.94–0.96
BMI	5.64	<0.05	1.20	1.03–1.38
Hypertension	5.84	<0.05	2.23	1.16–4.28
Major ECG abnormalities	12.04	<0.05	2.30	1.44–3.67

BMI, body mass index; CI, confidence interval; ECG, electrocardiogram; Se, selenium; VPC, ventricular premature complex.

## DISCUSSION

The results of the present study demonstrate that the presence of major ECG abnormalities among latent KD patients was associated with a higher risk of progression to chronic KD during the 10 years of follow-up compared to those with minor ECG abnormalities. Several previous studies have reported an association between ECG abnormalities and chronic KD outcomes in latent KD patients^15^; however, few studies have assessed reclassification with 10-year follow-up. Other publications on the ECG abnormalities of latent KD showed repolarization abnormalities, such as wide QRS/T angle, QT prolongation, and high QRS nondipolar voltage.^16^ Our findings are consistent with the aforementioned results.

The etiology of KD is not fully known, although several hypotheses have been suggested, including viral infection,^17^ intoxication with environmental toxicants or mycotoxins,^18^ and nutrition deficiency caused by a monotonous diet lacking minerals and vitamins such as molybdenum, magnesium, or thiamin.^19^^,^^20^ Among these potential etiologies, the selenium deficiency hypothesis has been considered most convincing. KD has been proven to be closely associated with selenium deficiency,^21^^,^^22^ which is associated with decreased activities of selenium-dependent antioxidant enzymes such as GPx. Other previous studies have reported that selenium deficiency can cause myocardial cell injury and ECG abnormalities in rats.^23^ In the present study, selenium deficiency was shown to be correlated with a decrease in GPx activity and was associated with the progression of latent KD to chronic KD (HR 0.95, 95% CI 0.94–0.96). Selenium supplementation has been shown to increase GPx activity, suggesting that selenium supplementation for latent KD patients with low blood selenium level can decrease the risk of progression to chronic KD.

VPCs are the most common form of ventricular arrhythmia. Assessment of VPCs is challenging and complex. A previous study showed that VPCs are correlated with an increased risk of heart failure during an average ± SD follow-up period of 15.6 ± 3.8 years.^24^ In the present study, newly occurring VPC abnormalities and other coexisting ECG abnormalities in latent KD patients during the follow-up were associated with an increased risk of subsequent progression to chronic KD and an increased risk of heart failure. This result suggests that VPCs may serve as a trigger for heightened awareness of and attention to cardiovascular risk assessment and management.

**Figure 3.**
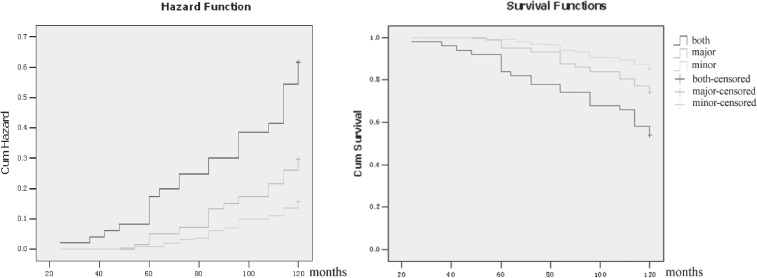
Kaplan-Meier estimates of chronic KD cumulative hazard over time of combined major vs minor and isolated major ECG abnormalities.

In the major ECG abnormalities group, 50 latent KD patients with combined ECG abnormalities had higher incidence of progression to chronic KD (23 patients, 46.0%) than those with isolated major abnormalities at baseline. After adjustment for baseline age, sex, family history of KD, initial cardiothoracic ratio, BMI, blood pressure, heart rate, smoking, and plasma selenium level, the HRs and 95% CIs for chronic KD events in latent KD patients with combined major ECG abnormalities and isolated major ECG abnormalities versus those with minor ECG abnormalities were 3.65 (95% CI 2.07–6.36) and 2.07 (95% CI 1.27–3.39), respectively (Figure 3). Using Cox proportional hazards regression models, we also found that atrial fibrillation and right bundle branch block with left anterior hemiblock are the most strongly predictive components of major ECG abnormalities (HR 13.73, 95% CI 5.34–52.33 and HR 26.1, 95% CI 3.52–134.86, respectively). Such results are consistent with previous studies.^8^ Moreover, some normal ECGs that converted from abnormal ECGs lasted less than 2 years before reverting to abnormal ECGs again. Some of patients who experienced this phenomenon progressed to chronic KD during the follow-up. Thus, latent patients with normal ECGs who previously had abnormal ECGs should have regular follow-up.

The areas where KD is endemic are all rural, and most are remote, mountainous, and difficult to access. Most KD patients are poor and disadvantaged with respect to utilizing health services; as such, echocardiography did not become routine in the diagnosis, management, and follow-up of KD patients until 2003. It has been shown that many latent KD patients have reasonably good function (New York Heart Association functional class I)^25^ but with an ECG abnormality at baseline. Annual or biennial ECG screening may be useful among latent KD patients. No particularly promising tool for predicting progression to chronic KD in latent KD patients has been identified from among the noninvasive screening methods studied to date. Thus, the safety, low cost, and wide availability of ECG are advantageous for use as an indicator for latent KD among high-risk patients. Further research should be conducted to establish a model with ECG and traditional risk factors or other novel promising biomarkers.

Previous studies had found that oral administration of selenite could significantly reduce the incidence of KD. One successful program for KD prevention has involved the supplementation of selenium salts to people in endemic areas, a practice which has been demonstrated to be a simple and effective way to prevent KD^26^; changes in people’s monotonous diets and improvement of the living standards of the people in the endemic areas is another way to reduce the incidence of KD. Furthermore, angiotensin-converting enzyme inhibitors and sodium selenite or organic selenium should be taken by latent KD patients with risk factors in order to delay or prevent progression to chronic KD.

### Limitations

There are several limitations to the present study. The sample size is small, thus lowering the statistical power. Nevertheless, the applied statistical method strengthened the results of the study. Only a single center in Shaanxi Province, northern China, was used in this study. Thus, the results may not be generalizable to similar latent KD patients in southern China. The ECG reading was not automated, and each was reviewed by 2 trained cardiologists, with discordant results adjudicated by a senior cardiologist. As previous literature shows large variations in accuracy of ECG readings in the clinical setting,^27^ precise estimation of how results may vary in the clinical setting is not possible. Reproducibility and reclassification using ECG may therefore be lower in the clinical setting than as we found. All latent KD patients were diagnosed according to the National Criteria for Diagnosis of Keshan Disease (China). If coronary artery disease is suspected in the patients living in endemic areas of KD, it is diagnosed and ruled out only by reviewing symptoms, medical history, risk factors, or further testing such as exercise ECG and echocardiography. Unfortunately, coronary angiography could not be performed in the local hospital due to limitations in study conditions.

### Conclusion

Major ECG abnormalities, specifically combined ECG abnormalities in the major ECG group, are associated with progression to chronic KD, and BMI and selenium deficiency are contributing factors at baseline. Hypertension and new VPC abnormalities that occurred in latent KD patients during later follow-up increased the risk of progression to chronic KD. Aggressive treatment of modifiable risk factors for chronic KD should be recommended for patients who are in higher risk categories. Future studies are required to investigate the feasibility of this suggestion.

In consideration of the safety, low cost, and wide availability of ECG, the ECG may be useful in improving chronic KD risk prediction in latent KD patients.

## References

[1] Ge K, Xue A, Bai J, Wang S Keshan disease—an endemic cardiomyopathy in China. Virchows Arch A Pathol Anat Histopathol. 1983;401(1):1–15 10.1007/BF00644785 6412443

[2] Tan J, Zhu W, Wang W, Li R, Hou S, Wang D, Selenium in soil and endemic diseases in China. Sci Total Environ. 2002;284(1–3):227–35 10.1016/S0048-9697(01)00889-0 11846167

[3] Kakehashi Y Why did Keshan disease occur? The relationship between White muscle disease, Shinshu cardiomyopathy and Keshan disease. Chin J Endemiology. 2000;19:150–2

[4] Li GS, Wang F, Kang D, Li C Keshan disease: an endemic cardiomyopathy in China. Hum Pathol. 1985;16(6):602–9 10.1016/S0046-8177(85)80110-6 3997137

[5] Yu PL Keshan disease: an entity or not. Hum Pathol. 1988;19:874 10.1016/S0046-8177(88)80275-2 3402979

[6] The State Technology Supervision Bureau and the Ministry of Health of the People’s Republic of China. Criteria for diagnosis of Keshan disease (GB17021-1997). National standards of the People’s Republic of China, Standards Press of China; 1998. 1–3.

[7] Wang T, Hou J, Zhang LJ National Keshan disease surveillance in 2007. Chin J Endemiology. 2008;118:412–5

[8] Xiang YZ The treatment and the management of patients with chronic Keshan disease should be enhanced in China. Chin J Endemiology. 2006;25:357–8

[9] Song HB Dynamic analysis of incidence and outcome of Keshan disease. Endemic Dis Bull. 1992;7:1–6

[10] The State Technology Supervision Bureau and the Ministry of Health of the People’s Republic of China. Delimitation and classification of Keshan disease areas (GB17020-1997). National standards of the People’s Republic of China, Standards Press of China; 1998.

[11] Prineas RJ, Crow RS, Blackburn HW. The Minnesota code manual of electrocardiographic findings: standards and procedures for measurement and classification[M]. Littleton, MA: J. Wright; 1982.

[12] Prineas RJ, Crow RS, Zhang ZM. The Minnesota Code Manual of Electrocardiographic Findings: Including Measurement and Comparison with the Novacode; Standards and Procedures for ECG Measurement in Epidemiologic and Clinical Trials[M]. Springer; 2009.

[13] Hershey JW, Oostdyk TS, Keliher PN Determination of arsenic and selenium in environmental and agricultural samples by hydride generation atomic absorption spectrometry. J Assoc Off Anal Chem. 1988;71:1090–3 3240958

[14] Kłapcińska B, Poprzęcki S, Danch A Blood selenium concentration of residents of Upper Silesia: relation to age and gender. Pol J Environ Stud. 2006;15:753

[15] Song H, Liang W, Yang Y Observation on clinical characters of latent keshan disease. J Xi’an Med Univ. 1992;13:384–6

[16] Xiang Y, Zhang W, Song S, Wang Z, Zhang H, Song J Clinical features and diagnosis of latent Keshan disease. Chin J Endemiology. 2001;20:304–7

[17] Li C, Niu X, Lei C Circulating adhesion molecules in patients with Keshan disease and their relationship with Coxsackie B virus infection. J Huazhong Univ Sci Technolog Med Sci. 2009;29(2):173–6 10.1007/s11596-009-0207-0 19399399

[18] Cermelli C, Vinceti M, Scaltriti E, Bazzani E, Beretti F, Vivoli G, Selenite inhibition of Coxsackie virus B5 replication: implications on the etiology of Keshan disease. J Trace Elem Med Biol. 2002;16(1):41–6 10.1016/S0946-672X(02)80007-4 11878751

[19] Yu WH A study of nutritional and bio-geochemical factors in the occurrence and development of Keshan disease. Jpn Circ J. 1982;46:1201–7 10.1253/jcj.46.1201 7131711

[20] Yang JB. A summary of epidemiological survey on Keshan disease (1965–1975): pathogenic role of mouldy food. In: Symposium of the 3rd China National Conference on Endemic Disease; 1995. p. 48–53.

[21] Yang GQ The relationship between selenium and etiology of Keshan disease. Sheng Li Ke Xue Jin Zhan. 1983;14:313–7 6678485

[22] Chen X, Yang G, Chen J, Chen X, Wen Z, Ge K Studies on the relations of selenium and Keshan disease. Biol Trace Elem Res. 1980;2(2):91–107 10.1007/BF02798589 24272892

[23] Toufektsian MC, Boucher F, Pucheu S, Tanguy S, Ribuot C, Sanou D, Effects of selenium deficiency on the response of cardiac tissue to ischemia and reperfusion. Toxicology. 2000;148(2–3):125–32 10.1016/S0300-483X(00)00203-1 10962131

[24] Agarwal SK, Simpson RJ Jr, Rautaharju P, Alonso A, Shahar E, Massing M, Relation of ventricular premature complexes to heart failure (from the Atherosclerosis Risk In Communities [ARIC] Study). Am J Cardiol. 2012;109(1):105–9 10.1016/j.amjcard.2011.08.009 21945138 PMC3242884

[25] Wei Y, Liu B, Zhu J, Yang J, He X, Niu X Tei Index Evaluation of Ventricular Function in Patients with Keshan Disease. Chin J Ultrasound Med. 2006;22:914–6

[26] Cai W, Xia W, Deng J, Hu W Analysis of effect of selenium-salt on preventing Keshan disease in Liangshan state in 2001. Chin J Endemiology. 2003;22:439–40

[27] Salerno SM, Alguire PC, Waxman HS Competency in interpretation of 12-lead electrocardiograms: a summary and appraisal of published evidence. Ann Intern Med. 2003;138:751–60 10.7326/0003-4819-138-9-200305060-00013 12729431

